# Assessment Study on the Solvent Resistance of Low-Density Polyethylene with Pumpkin Seed Hulls

**DOI:** 10.3390/ma16010138

**Published:** 2022-12-23

**Authors:** Karolina Głogowska, Przemysław Pączkowski, Barbara Gawdzik

**Affiliations:** 1 Department of Technology and Polymer Processing, Faculty of Mechanical Engineering, Lublin University of Technology, Nadbystrzycka 36, 20-618 Lublin, Poland; 2 Department of Polymer Chemistry, Institute of Chemical Sciences, Faculty of Chemistry, Maria Curie-Skłodowska University in Lublin, Gliniana 33, 20-614 Lublin, Poland

**Keywords:** hybrid injection-moulded parts, natural fillers, low-density polyethylene, solvent

## Abstract

When designing products that are made of composite materials and that contain natural fillers, it is particularly important to consider the long-term exposure of these materials to caustic liquids and substances (concentrated acids, bases), and to ensure that these products meet strict requirements for reliability and operational safety. This study investigated the effects of different solvents on the mass, mechanical, thermal, surface, and structural properties of polymer composites containing natural fillers in the form of pumpkin seed hulls. Experiments were conducted using four different filler contents (5, 10, 15, and 20 wt%) and grain sizes ranging from 0.2 to 0.4 mm and 0.6 to 0.8 mm. Hybrid injection-moulded pieces were immersed in distilled water (H_2_O), 1% NaOH solution, acetone (C_3_H_6_O), and toluene (C_7_H_8_) for 84 days. After that, their mechanical and thermal properties as well as their structure geometries were analysed statistically. Changes in the properties of the tested composite materials were assessed depending on the solvent type, and the statistical significance of these changes were determined. The results showed that the nature of degradation depended on the applied solvent type. It was observed that the polymer matrix of the toluene-immersed samples absorbed the liquid to a significant extent first and then underwent a gradual degradation with time. In contrast, the samples immersed in water showed a slight increase in their mass. It was found that all tested properties of the composite samples deteriorated irrespective of the solvent type.

## 1. Introduction

Environmental concerns have prompted research on the use of sustainable materials. The most widely discussed sustainability parameters pertain to the replacement of traditional materials with their alternatives. As a result, industries have invested in new techniques and technologies and included environmental protection aspects in their production strategies [1].

The interest in adding natural fibres to the polymer matrix has had a profound impact on reducing the need for materials from non-renewable sources. In addition to that, this has also had an effect on the related environmental and economic aspects. Natural fibres are attractive materials because of their biodegradability, low density, and abrasion, not to mention the fact that their mechanical properties are comparable to those of fibre glass-reinforced composites [2].

What prompts the use of natural fibres as composite fillers is the prospect of saving energy by reducing product mass, along with the aspects related to the recovery of raw materials and material recycling at the end of a product’s life cycle. Furthermore, the scale of agro-industrial waste being generated these days is enormous; hence, the idea of waste material reusing seems interesting.

Polyethylene is a high molecular mass hydrophobic polymer that takes 100 years to degrade in the environment [3]. Similarly to other thermoplastics, it is used in large quantities for low-cost applications. Therefore, the production of natural fibre-reinforced composites is an important alternative to recycling polyethylene (which is the most widely used thermoplastic in the world) and can be a solution to the polymer waste pollution of water systems and inland environments. Low-density polyethylene (LDPE) has been widely used as the matrix material in composites reinforced with natural fibres, such as sugar beet pulp [4], pineapple leaf fibre [5], pomelo foam powder [6], different types of starches [3,7,8], peanut husk [9], wood flour [10], palm fibres [11], sunflower husk waste [12], and wheat bran [13].

It is known that oxygen in the air, high temperatures, hydrolysis caused by moisture, light wavelength (>300 nm), high-energy radiation, such as UV, mechanical stress, biological attacks, and contact with aggressive liquids and some living organisms, have detrimental effects on polymeric materials [14,15,16].

In semi-crystalline polymers, such as polyethylene, the aging process affects the amorphous fraction at an early stage. This is due to molecular relaxations induced by an external stimulus to disordered polymer chains. However, the crystallinity decreases as the material ages, which also indicates the degradation of the crystalline fraction of the polymer. The properties of semi-crystalline polymers depend on the ratio of the crystalline fraction to the amorphous fraction. Therefore, it is believed that the crystalline fraction has a direct effect on the polymeric material properties in the aging process [17]. On the other hand, it is important to bear in mind that natural fillers also undergo aging. This occurs when the filler components, such as cellulose, hemicellulose, and lignins degrade individually or when interfacial bonds are broken [18,19]. Therefore, it is difficult to predict without prior testing whether reinforced composites would retain their properties.

Despite the numerous advances in the development of polymer composites, there are very few studies on the behaviours of their properties under simulated usage conditions, such as immersing them in different solvents.

The objective of this study was to fabricate low-density polyethylene composite samples containing natural fillers in the form of ground pumpkin seed hulls and to determine how immersing these samples in different solvents would affect their physicochemical, mechanical, and geometrical properties.

## 2. Materials and Methods

### 2.1. Materials

Test specimens were made of powdered low-density polyethylene (LDPE) Dowlex 2631.10UE manufactured by DOW Chemical (Schkopau, Germany). This material is used to produce thin-walled parts by rotational casting and high-accuracy parts via injection moulding. Table 1 lists the properties of this material according to the specifications given by the manufacturer.

**Figure 1:**
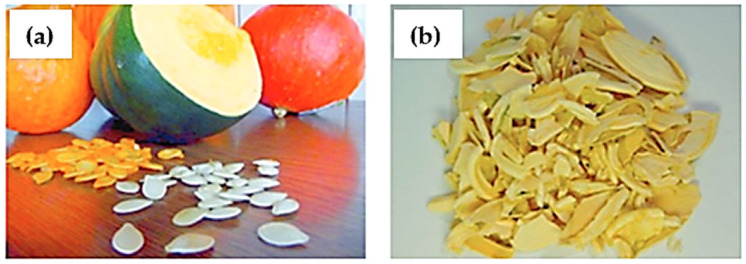
General view of (**a**) pumpkins and their seeds and (**b**) pumpkin seed hulls.

A growing interest in environmental protection has resulted in numerous studies devoted to advanced materials made of natural raw materials, such as polymer–matrix composites. The production of materials based on renewable and natural waste materials has become an area of interest for many scientists dealing with material engineering and polymer processing across the world. In this study, pumpkin seed hulls were used as the natural fillers (Figure 1). They were obtained from a plant-based near Lublin, Poland, which cleans and sells pumpkin seeds. Hulls are waste products derived from the mechanical peeling and cleaning of pumpkin seeds. The main components of pumpkin seed hulls are mixtures of polysaccharide (cellulose, hemicellulose, pectin, mucilage) and non-polysaccharide (lignin) substances [1].

### 2.2. Methods

**Figure 2:**
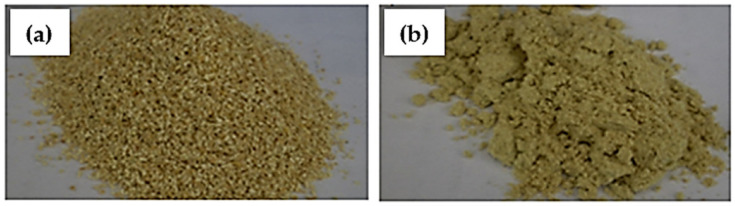
View of pumpkin seed hull fractions with their grain sizes ranging from (**a**) 0.6 to 0.8 mm and (**b**) 0.2 to 0.4 mm.

The organic filler was prepared by grinding pumpkin seed hulls into powder and then separating fractions of specific grain sizes using sieves with mesh sizes of 0.4 mm and 0.8 mm (Figure 2). As a result, two fractions were obtained, with their grain sizes ranging from 0.2 to 0.4 mm and 0.6 to 0.8 mm.

The conditioning process for low-density polyethylene and pumpkin seed hulls consisted of drying the composition for 24 hours at 70 °C in a laboratory dryer. Prior to injection moulding, the feedstock was mechanically dry-mixed with a planetary mixer. A loose mixture was produced, and it was poured directly into the hopper of the injection moulding machine. The prepared samples of low-density polyethylene filled with pumpkin seed hulls had different mass percentages of 5, 10, 15, and 20%, and grain sizes ranging from 0.2 to 0.4 mm and 0.6 o 0.8 mm.

Experiments were performed on the Arburg Allrounder 320C single-screw injection moulding machine (Arburg, Loßburg, Germany), equipped with a two-cavity mould for manufacturing standard samples in accordance with ISO 294-1:2017-07 [20]. The dumbbell samples had a total length of 150 mm and a thickness of 4 mm; the width of the measuring part was 10 mm and that of the grip part was 20 mm. The injection moulding process parameters were the same for all tested polymeric compositions. Individual zones of the plasticizing system had the following temperatures: I—130 °C, II—135 °C, III—140 °C, IV—150 °C, and the temperature of the injection mould was 30 °C. The injection time was set to 2 s, and the cooling time was 30 s.

For the purpose of this study, the fabricated samples were described as follows: solvent molecular formula/natural filler content/grain size/tested polymeric material symbol, e.g., H_2_O/5/0.4/LDPE means that the sample was immersed in distilled water and contained 5 wt% natural filler with a grain size of 0.2–0.4 mm.

The behaviour of pure LDPE and its composites with different filler contents and grain sizes following immersion in different liquid chemicals was investigated in accordance with the EN ISO 175:2010 standard [3]. The cuboid-shaped samples were put into separate airtight containers with 50 mL of a given liquid and then stored in a darkened place at ambient temperature (23 ± 2 °C). Their chemical resistance was tested for distilled water (H_2_O), 1% NaOH solution, acetone (C_3_H_6_O), and toluene (C_7_H_8_). From time to time, the samples were taken out from the solvents, rinsed with distilled water, and gently wiped. The mass change Δ*m* was calculated using Equation (1):

(1) $$\Delta m=\frac{m_{i}-m_{0}}{m_{0}}\times 100\%$$

where *m*_0_ is the initial mass of a dry sample (g) and *m_i_* is the mass of a sample after some time of the solvent immersion (g).

The strength properties, such as flexural strength and flexural modulus, were determined in compliance with the ISO 178:2019 standard [21]. The tensile test was conducted with a speed of 20 mm min^−1^. The test was performed on a universal testing machine, Zwick Roell Z010 (Ulm, Germany).

The hardness of the samples was measured by the Shore method using an ART.13 device from Affri System Hardness Testers (Induno Olona, Italy). The measurements were conducted in compliance with the procedure described in ISO 527-1:2012 [22].

The Vicat softening temperature (VST) was measured using Ceast HV3 from Instron (Turin, Italy) in compliance with ISO 306:2013 [23]. The measurements were conducted by the A120 method, with a load of 10 N and a heating rate of 120 °C h^−1^.

The microstructure of the samples was examined using a high-resolution digital microscope from Keyence (Mechelen, Belgium).

Surface geometry was examined per four specimens from each type of fabricated injection-moulded piece. The examination was performed with the use of a device for measuring the contour, roughness, and 3D topography from Hommel-Etamic (Jena, Germany). The following surface roughness parameters were measured: *Ra*, *Rz*, *Rmax*, *Rq*, and *RSm*. The measurements were made in compliance with the ISO 11652:1996 standard [24]. The measuring range was 400 µm, and the mapping distance was set to equal 4.80 mm. The measurements were conducted at a speed of 0.80 mm s^−1^.

## 3. Statistical Analysis

The experimental results are presented as mean values and standard deviations, in compliance with the recommendations specified in ISO: 178, 527-1, and 306. However, given the fact that this approach would not provide sufficient data to draw conclusions about statistically significant differences between individual sample batches, statistical analyses were additionally performed for a significance level of α = 0.05 in order to estimate the actual changes in flexural strength, flexural modulus, hardness, and Vicat softening point. The analyses were conducted using the data analysis software system Statistica version 13.3 from TIBCO Software, Inc. (2017) (Palo Alto, CA, USA). The use of the multiple comparisons method for mean values obtained in several groups to account for the differences detected by the variance analysis makes it possible to group the mean values and, thus, extract homogeneous groups, i.e., the groups of mean values that do not differ statistically from each other. To that end, Tukey’s test was selected from the available solutions (e.g., Scheffé, Tukey, Newman–Keuls, Duncan, and Fisher tests).

## 4. Results and Discussion

### 4.1. Immersion-Induced Degradation

As previously mentioned, polymer degradation depends on the combination of destructive factors, such as contact with microorganisms, chemical agents, air, humidity, temperature, light, high-energy radiation, and mechanical stresses. These factors cause irreversible changes in the properties of polymeric materials, an effect that can be relatively quickly observed for thermoplastics, whereas cross-linked polymers show some resistance to these destructive factors. This study aims to determine the influence of immersing low-density polyethylene-based composite samples in various solvents on the mechanical properties of these samples.

Many different effects of solvents on polymers have been described in the literature. They can be used for porogen extraction from a porous Cu/LDPE composite [25], for the transition from mushroom to brush conformation for PEG grafting to highly curved TiO_2_ NPs, [26] or for the carbodiimide-mediated cross-linking of gelatine nanofiber [27]. It is generally believed that solvents are insoluble in the crystallite regions of the polymeric material and that the mass transfer or diffusion only occurs in the continuous amorphous phase between the crystallites.

There is a correlation between the free volume of the amorphous parts in polyethylenes and their crystallinity [28]. A higher free volume occurs in the amorphous phase of LDPE. Both diffusivity and penetrating solubility in the amorphous polymer phase decrease with increasing crystallinity. Moreover, the tortuosity or length of the diffusion path around the crystals increases with the degree of crystallinity. Thus, the sorption properties using organic solvents will give an idea about the internal structure of the polymer. 

The interactions between polymer solvents with low-density polyethylene composites depend on various factors, such as the crystallinity and polarity of the fillers, their adhesion and compatibility with the polymer matrix, the size and nature of the penetrants, etc. [29].

One important parameter of composites containing hydrophilic fillers concerns their water uptake abilities. In general, as the water content in the composite increases, all functional properties of a product decrease, which can be explained by the breakage of adhesion between the matrix and the filler particles as well as the formation of micropores and microcracks [18].

**Figure 3:**
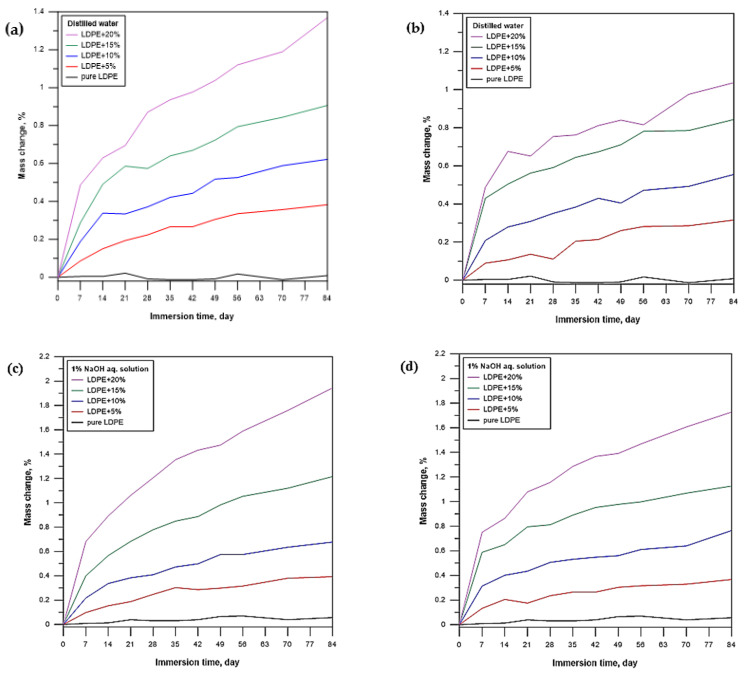

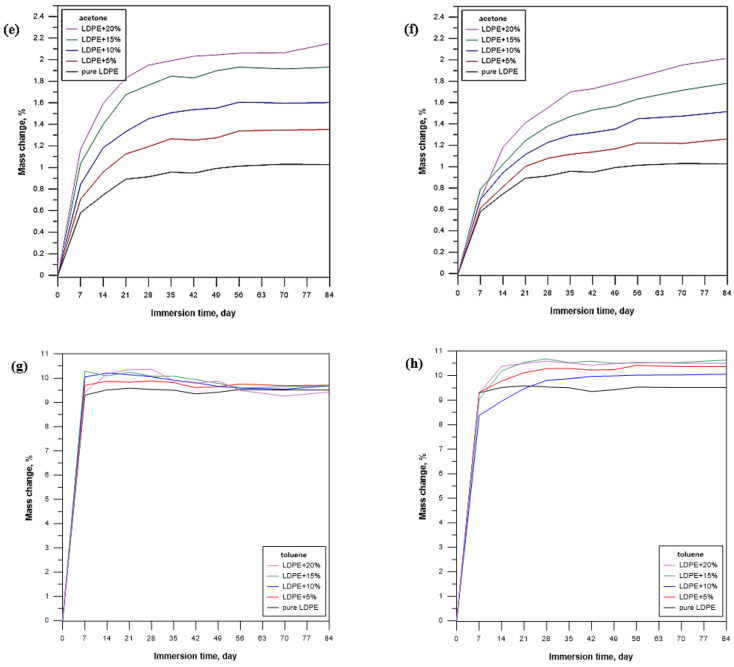
Mass change in pure LDPE and LDPE with a natural filler versus different solvents: (**a**) distilled water—filler grain size: 0.2–0.4 mm, (**b**) distilled water—filler grain size: 0.6–0.8 mm, (**c**) NaOH solution—filler grain size: 0.2–0.4 mm, (**d**) NaOH solution—filler grain size: 0.6–0.8 mm, (**e**) acetone—filler grain size: 0.2–0.4 mm, (**f**) acetone—filler grain size: 0.6–0.8 mm, (**g**) toluene—filler grain size: 0.2–0.4 mm, (**h**) toluene—filler grain size: 0.6–0.8 mm.

Figure 3a–h show the relationship between the composite sample mass and immersion time. The figures show examples of curves obtained for the samples that were immersed for 84 days in water, NaOH, acetone, and toluene. All curves are shown in the same order. Following the immersion test, the pure LDPE sample shows the smallest mass change. The samples of LDPE filled with pumpkin seed hulls exhibit the largest mass gain. In general, all composite samples behave similarly in the tested aqueous solutions. Moreover, it can be observed that grain size affects solvent absorption. Regarding the samples immersed in water and NaOH solution, the smaller particles of the filler (0.2–0.4 mm) cause the material to absorb the solvents more readily. For acetone and toluene, the solvent uptake is on a fairly similar level and does not depend on the filler content or grain size, but rather on the polymeric matrix itself.

Water molecules can act as natural plasticizers and, thus, make the filler flexible, as opposed to it being hard and rigid in a completely dry state [8,9].

The content of pumpkin seed hulls impacted the water absorption properties of the samples. This effect can be observed in Figure 3a,b, showing the variation in water sorption during immersion. Water absorption increases with the immersion time and filler content. For some samples, rapid water absorption can be observed during the first few days of the solvent immersion, followed by an equilibrium state over time. Water absorption is related to the rate of water diffusion into the composite samples. Synthetic materials based on pumpkin seed hulls tend to absorb water because the hydroxyl group present in the filler can form a hydrogen bond with water. Hence, the hydrophilic nature of the filler tends to attract water molecules [19].

In addition, water molecules can easily saturate the surface of the polyethylene/pumpkin seed hull composites as well as penetrate the composites through voids, which results in their higher water absorption over a shorter exposure time.

The absorption of the NaOH solution (Figure 3c,d) is similar to that observed for distilled water, which is due to the impact of the lignocellulosic filler. For both cases, the solvent uptake by the pure polyethylene samples is negligible. This is due to the chemically hydrophobic nature of the polymeric matrix.

A totally different situation can be observed for the samples immersed in organic solvents (Figure 3e–h). An analysis of the curves reveals that absorption primarily affects the polymer matrix rather than the filler itself.

Following the contact with acetone, the mass of the pure LDPE samples increased by about 1%. Similar to water, acetone absorption increased with the immersion time and filler content, and the equilibrium state was reached after about 49 days. This phenomenon was not observed for the composite samples with higher grain sizes even until the 84th day of the test.

The most interesting observations were made for the pure LDPE samples and the composites that were immersed in toluene. After 7 days, a sharp 9–10% increase in the mass of these samples was observed. For this case, it is difficult to unanimously state whether the filler had a direct impact on the solvent absorption; nevertheless, a gradual decrease in the mass of the composite was more clearly observed after 35 days. The polyethylene matrix undergoes degradation by partial dissolving in the aggressive environment [30]. Polymeric material dissolution is sometimes considered a type of chemical recycling. This phenomenon was not observed for the composite samples with larger grain sizes (0.6–0.8 mm). For this case, the toluene absorption was also about 10% and lasted a little longer, and the state of equilibrium was not achieved until the 84th day of the immersion test.

### 4.2. Mechanical Properties

To compare the behaviours of the samples immersed in different solvents, their flexural strengths, moduli of elasticity at bending, and hardness were used as the measures of immersion-induced degradation.

#### 4.2.1. Strength Properties

**Figure 4:**
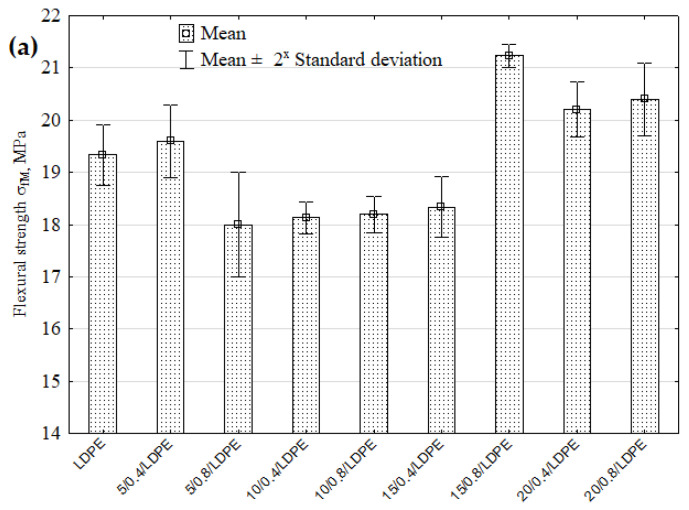

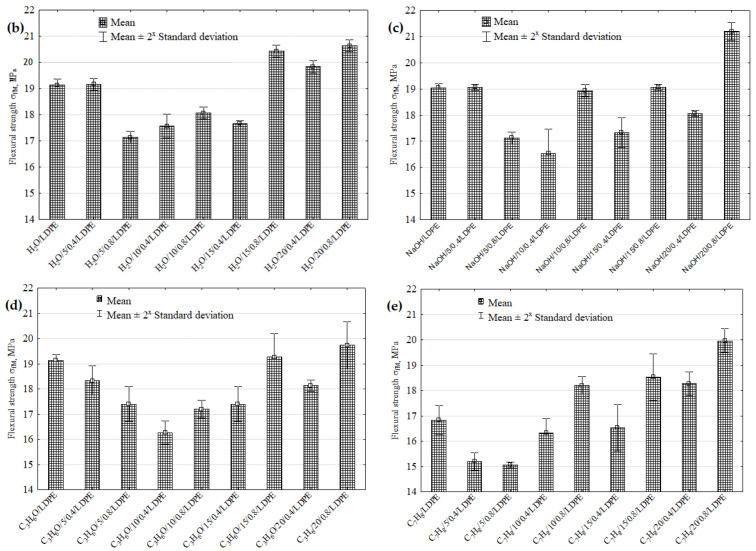
Flexural strength of pure LDPE and LDPE with a natural filler versus different solvents: (**a**) reference samples, (**b**) distilled water, (**c**) NaOH solution, (**d**) acetone, (**e**) toluene.

Figure 4a–e show the flexural strength results obtained for the samples of low-density polyethylene with natural fillers before and after the solvent immersion. The results demonstrate that the LDPE samples that contained at least 15 wt% natural filler with a fraction of 0.6–0.8 mm increased their flexural strength by 9.82% (compared to the reference LDPE samples). The statistical analysis showed significant differences between the flexural strength values of 5/0.8/LDPE–15 /0.8/LDPE samples (Figure 4a). Nourbakhsh et al. [31] observed that composites made from poplar fibres exhibited improved strength properties as a result of more fibre-to-fibre and fibre–plastic contact. They also showed that the strength properties increased with the fibre aspect ratio. Some researchers have demonstrated that the strength properties of composite materials decrease with particle size reductions.

A comparison of the flexural strength results obtained for the samples after the solvent immersion reveals that their strength decreased irrespective of the solvent type (Figure 4b–e). Compared to the reference LDPE samples, the highest strength decrease (by 21.88%) can be observed for the toluene-immersed samples with 5 wt% filler content and grain sizes of 0.6–0.8 mm (Figure 4e). The Kruskal–Wallis test showed significant differences between the flexural strength values of the following pairs of samples: H_2_O/5/0.8/LDPE–H_2_O/5/0.8/LDPE, H_2_O/5/0.8/LDPE–H_2_O/20/0.8/LDPE (Figure 4b), NaOH/10/0.4/LDPE–NaOH/20/0.8/LDPE (Figure 4c), C_3_H_6_O/10/0.4/LDPE–C_3_H_6_O /20/08/LDPE (Figure 4d), C_7_H_8_O/5/0.4/LDPE–C_7_H_8_O/20/0.8/LDPE, and C_7_H_8_O/5/0.8/LDPE–C_7_H_8_O/20/0.8/LDPE (Figure 4e).

Previous studies investigated the effects of different natural fillers on selected properties of polymer composites, showing that the flexural strengths of these polymer composites increased with the increasing lignocellulosic filler content [4,32,33,34].

**Figure 5:**
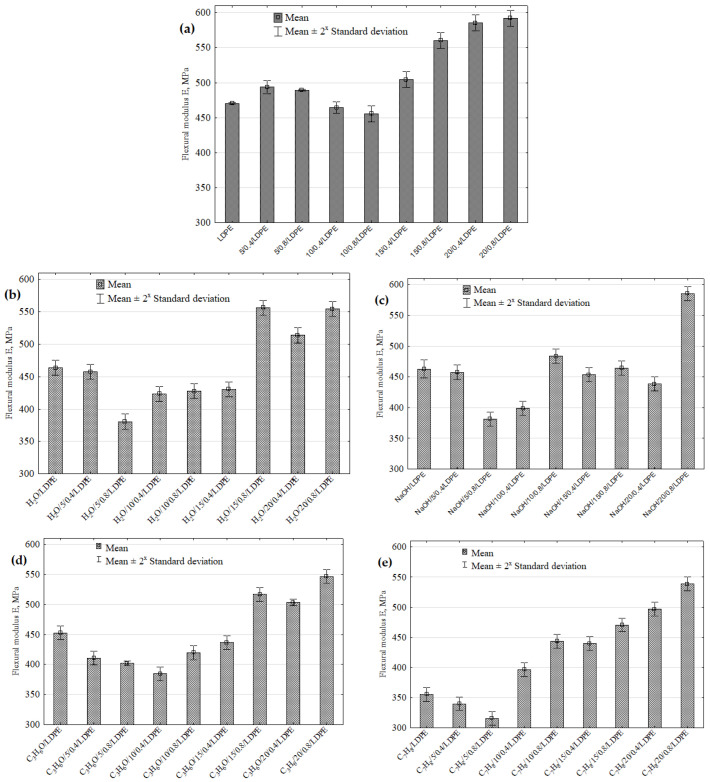
Flexural modulus of pure LDPE and LDPE with a natural filler versus different solvents: (**a**) reference samples, (**b**) distilled water, (**c**) NaOH solution, (**d**) acetone, (**e**) toluene.

Flexural modulus results obtained for the samples before and after immersion-induced degradation are shown in Figure 5a–e. The results demonstrate that the flexural modulus increased by 25.82% in the 20/0.8/LDPE samples compared to the reference samples made of pure LDPE. The statistical analysis results showed significant differences between the mean flexural moduli of the following pairs: 10/0.8/LDPE–20/0.4/LDPE and 10/0.8/LDPE–20/0.8/LDPE (Figure 5a). Generally, it can be claimed that the mechanical properties of the polymer composites depend on the filler type, volume fraction, arrangement, and grain size.

An analysis of the flexural modulus results reveals that this property decreases in the samples after the solvent immersion, irrespective of the solvent type (Figure 5b–e). The highest decrease in the flexural modulus amounting to 21.88% can be observed for the toluene-immersed samples with a 5 wt% filler content and a grain size of 0.6–0.8 mm (Figure 4e). The multiple comparisons test showed significant differences between the flexural moduli of the following pairs of samples: H_2_O/5/0.8/LDPE–H_2_O/15/0.8/LDPE, H_2_O/5/0.8/LDPE–H_2_O/20/0.8/LDPE (Figure 5b), NaOH/5/0.8/LDPE–NaOH/10/0.8/LDPE, NaOH/5/0.8/LDPE–NaOH/20/0.8/LDPE (Figure 5c), C_3_H_6_O/10/0.4/LDPE–C_3_H_6_O/20/08/LDPE, C_3_H_6_O/5/0.8/LDPE–C_3_H_6_O/20/08/LDPE (Figure 5d), C_7_H_8_O/5/0.4/LDPE–C_7_H_8_O/20/0.8/LDPE, C_7_H_8_O/5/0.8/LDPE–C_7_H_8_O/20/0.4/LDPE, and C_7_H_8_O/5/0.8/LDPE–C_7_H_8_O/20/0.8/LDPE (Figure 5e). Composite products made of two or more constituent materials are more sensitive to solvents and humidity than homogenous products. This relationship can be observed for three of the tested liquids: water, NaOH solution, and acetone. For the toluene-immersed LDPE samples, their flexural moduli values significantly decreased from 470.5 to 355.6 MPa, which amounted to 25.42%. This decrease was probably caused by a rapid 9–10% increase in the mass of the toluene-immersed samples (Figure 3h).

#### 4.2.2. Hardness

**Figure 6:**
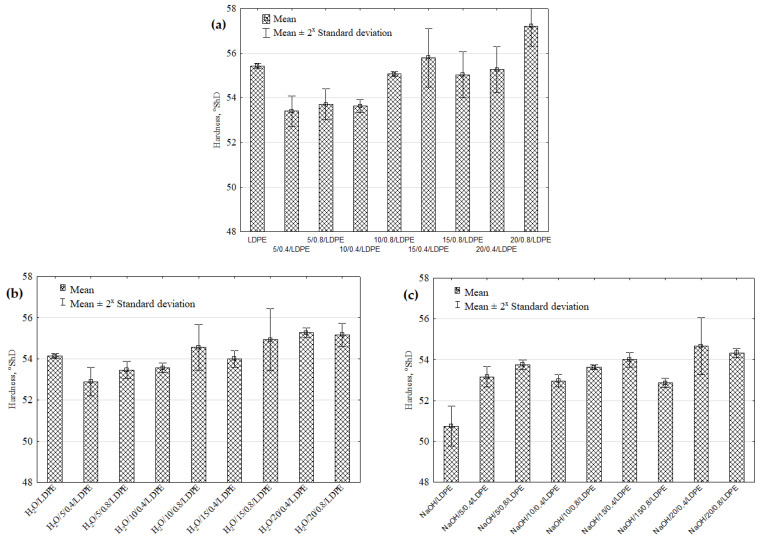

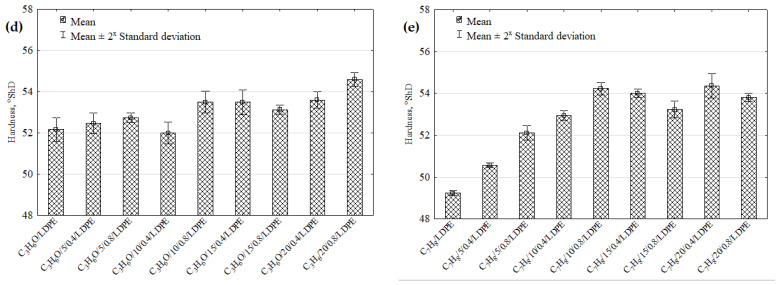
Hardness of pure LDPE and LDPE with a natural filler versus different solvents: (**a**) reference samples, (**b**) distilled water, (**c**) NaOH solution, (**d**) acetone, (**e**) toluene.

The hardness results of the hybrid injection-moulded samples are shown in Figure 6a–e. The highest hardness can be observed for the samples with 20 wt% filler content and grain sizes of 0.6–0.8 mm; their mean hardness was 57.2 °ShD. This may be associated with their increased stiffness due to the presence of more fillers in the matrix [9].

The lowest hardness of 53.4 °ShD was obtained for the samples with a 5 wt% filler content and grain sizes of 0.6–0.8 mm. The statistical analysis results demonstrate that for the assumed significance level of α = 0.05, there exist statistically significant differences for two groups of the samples: 5/0.4/LDPE–20/0.8/LDPE and 10/0.4/LDPE–20/0.8/LDPE (Figure 6a).

The hardness results obtained for the samples after the immersion reveal that their hardness decreases whatever the solvent type (Figure 6b–e). The lowest hardness of 49.13 °ShD was obtained for the unfilled low-density polyethylene samples immersed in toluene. Compared to the reference LDPE sample, the hardness decreased by 11.18%. The statistical analysis results showed statistically significant differences for individual groups of the samples, namely: H_2_O/5/0.4/LDPE–H_2_O/20/0.4/LDPE (Figure 6b), C_3_H_6_O/LDPE–C_3_H_6_O/20/0.8/LDPE, C_3_H_6_O/10/0.4/LDPE–C_3_H_6_O/20/0.8/LDPE (Figure 6d), C_7_H_8_O/LDPE–C_7_H_8_O/10/0.8/LDPE, and C_7_H_8_O/LDPE–C_7_H_8_O/20/0.4/LDPE (Figure 6e). 

Generally, polymeric materials can absorb liquids from the environment. The reduction in the mechanical properties of the materials tested in this study was probably caused by both hydrolytic degradation and osmotic cracking [16]. In osmotic cracking, an osmotic pressure is generated inside the polymer matrix by the diffusion of liquid molecules, which may lead to microcrack nucleation and, thus, to reduced material properties.

### 4.3. Vicat Softening Temperature (VST)

**Figure 7:**
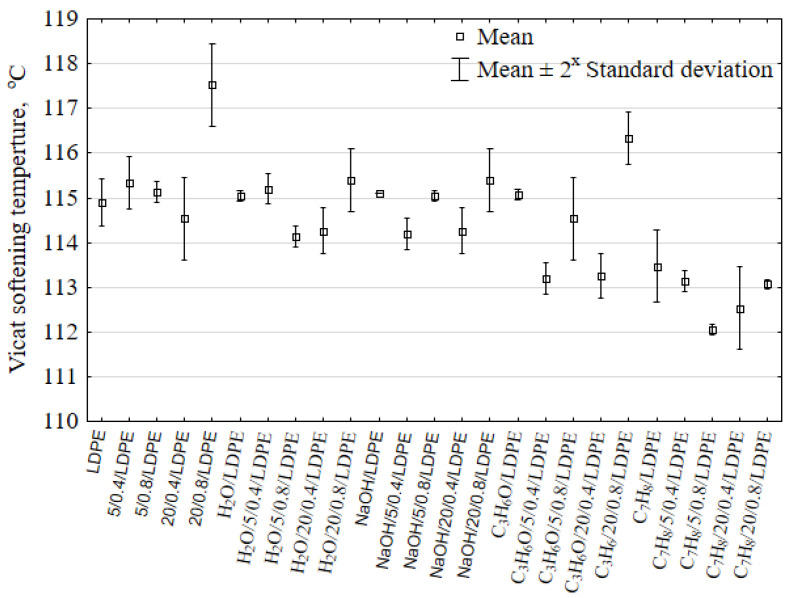
Vicat softening temperature of the selected samples of pure LDPE and LDPE with natural fillers versus different solvents.

A relationship between the Vicat softening temperature and the filler content/particle size as well as the solvent type is plotted in Figure 7. The plot only shows the extreme filler contents, i.e., 5 and 20 wt%. As a result of loading the 20 wt% filler content into the LDPE matrix with a softening temperature of 114.9 °C, the softening temperature value increased to 117.53 °C. There exists no direct dependence to describe the effect of powder fillers on the Vicat softening temperature. Changes in this temperature primarily depend on the interaction between the filler and the polymer matrix. For the analysed case, it is difficult to claim that the filler impacted the softening temperature due to the fact that the percentage variations in this temperature did not exceed 2.28% of the initial value.

The softening temperature results obtained for the samples after the solvent immersion demonstrate that the Vicat softening temperature decreases regardless of the solvent type. The lowest temperature of 112.6 °C was obtained for the toluene-immersed hybrid injection-moulded part with a 5 wt% filler content and a grain size of 0.6–0.8 mm. The multiple comparisons test showed significant differences between the softening temperature values of the following groups: 20/0.8/LDPE–C_7_H_8_O/20/0.4/LDPE, 20/0.8/LDPE–C_7_H_8_O/5/0.8/LDPE, and C_7_H_8_O/5/0.8/LDPE–C_3_H_6_O/20/0.8/LDPE. The solvent molecules can act as natural plasticizers and, thus, cause matrix plasticization, which has a negative effect on the mechanical and thermal properties of composite materials.

### 4.4. Surface Roughness

Surface roughness parameters are used for evaluating the geometric structure of a given surface. In this study, the following surface roughness parameters were selected for the measurements: *Ra*—mean roughness (i.e., the arithmetic average of the absolute values of the roughness profile ordinates); *Rz*—maximum height of the profile; *Rmax*—maximum roughness depth; *Rq*—root mean square deviation of the roughness profile; *RSm*—mean spacing of the profile elements. The surface roughness of the injection-moulded 20/0.8/LDPE samples was measured before and after the solvent immersion. The obtained surface roughness results are listed in Table 2.

The surface roughness results demonstrate that all tested parameters, i.e., *Ra*, *Rz*, *Rmax*, *Rq*, and *RSm,* increased compared to the results obtained for the samples prior to the solvent immersion. The liquids reduced the surface quality of the samples. The highest values of the surface roughness parameters were obtained for the toluene-immersed samples. 

**Figure 8:**
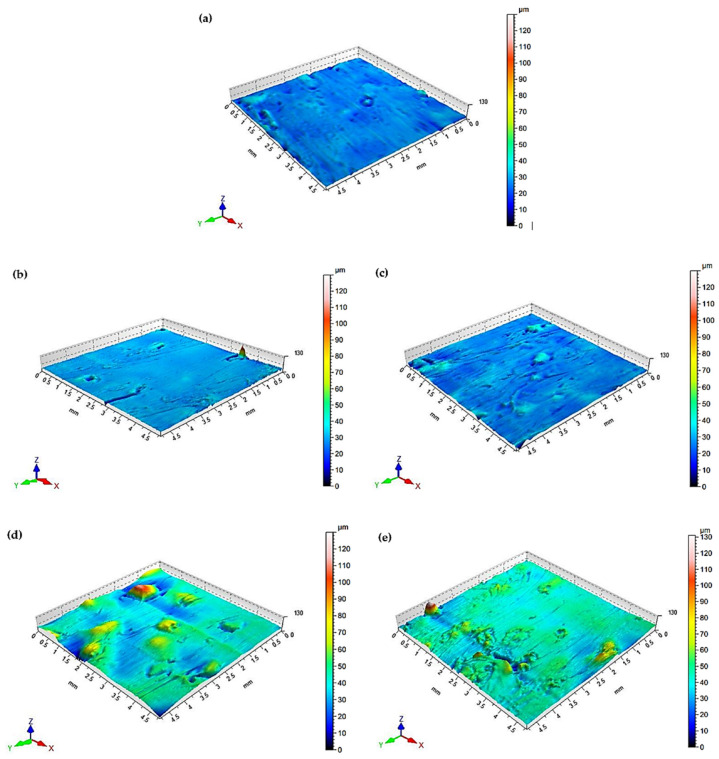
Surface topography of polymer composite samples containing 20 wt% natural filler, i.e., pumpkin seed hulls with grain sizes of 0.6–0.8 mm (the samples are shown before and after the solvent immersion); (**a**) reference sample, (**b**) distilled water, (**c**) NaOH solution, (**d**) acetone, (**e**) toluene.

The polymer composite samples were also examined for their surface topographies. Figure 8a–e show examples of the surface topography of the 20/0.8/LDPE samples before and after the solvent immersion. An analysis of the surface topographies reveals that the C_7_H_8_O/5/0.8/LDPE samples had the worst surface quality compared to those obtained for 20/0.8/LDPE. The presence of the yellow–green areas is stronger, which indicates higher surface roughness heights.

### 4.5. Microscopic Structure

**Figure 9:**
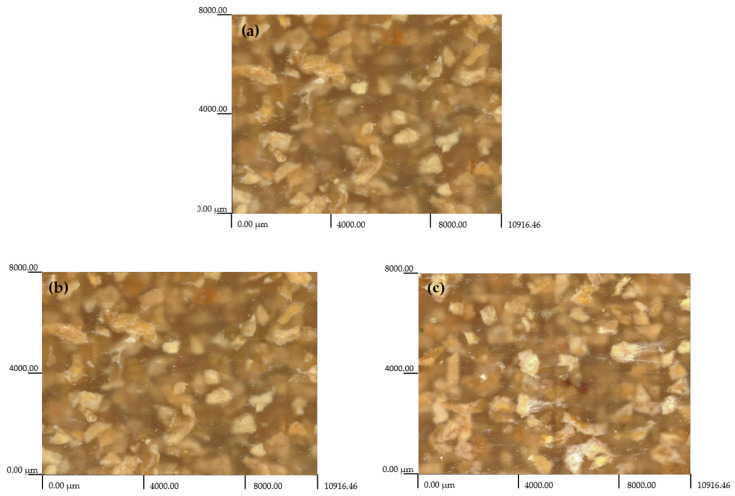

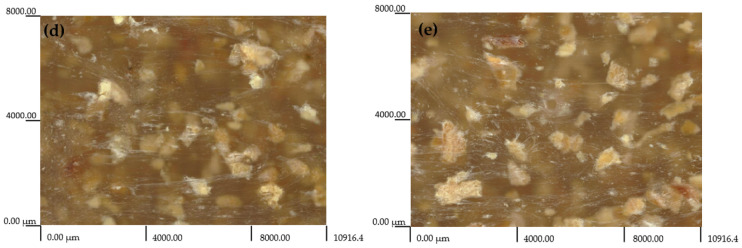
Microphotographs of polymer composite samples containing 20 wt% natural filler, i.e., pumpkin seed hulls with grain sizes of 0.6–0.8 mm (the samples are shown before and after the solvent immersion): (**a**) reference sample, (**b**) distilled water, (**c**) NaOH solution, (**d**) acetone, (**e**) toluene.

In this paper, we present images of the morphologies of the tested injection mouldings with 20 wt% filler content and grain sizes of 0.6–0.8 mm (Figure 9a–e), with a special focus on surface defects. An analysis of the structure shows that there is a non-uniform distribution of the natural filler. There are particles of varying sizes, irregular shapes, and agglomerates, which indicates that the structure is not homogeneous. The micrometric pumpkin seed hull particles likely have higher surface energy than low-density polyethylene and, thus, have a tendency to agglomerate in this medium. This effect is undesirable because it reduces the contact area between the filler and the polymer, leading to decreased adhesion, which is conducive to the formation of agglomerates. An analysis of the images also leads to the conclusion that the grounded pumpkin seed hull grains are not uniform in terms of their shapes and dimensions, which is most likely due to the shapes of pumpkin seed hulls and their mechanical properties. The grains formed as a result of mechanical grinding are plate-shaped, with the one dimension larger than the size range adopted in this study. This demonstrates that the resulting polymer mixture must be thoroughly mixed in order to obtain a homogeneous structure and, thus, the desired properties of the resulting product.

## 5. Conclusions

The modification of polymeric plastics by adding various types of natural fillers causes many changes in their processing, mechanical and thermal properties, as well as final product morphology. Regarding the applications of polymeric compositions, it is important that the changes in the properties of modified plastics be examined. It is also very important to investigate how given products will behave under different operating conditions. This study attempted to determine the effects of immersing pumpkin seed hull-filled LDPE composite samples into four different liquids.

The study showed that polyethylene composites behaved differently when immersed in different solvents. The natural fillers (pumpkin seed hulls) had significant impacts on the absorption of distilled water and the NaOH solution. The filler quantity and particle size proved to be equally important. By increasing the pumpkin seed hull content in the material, the solvent absorption increased too. A similar trend was observed for the filler with a larger grain size, yet the change in mass was smaller.

The organic solvents (acetone and toluene) were found to interact more readily with the polymer matrix than with the filler. Toluene proved to be the most aggressive environment for pure LDPE and its composites filled with pumpkin seed hulls. There was a significant increase in the composite mass over a very short period of time, which was followed by a gradual mass decrease after some time. This was due to the polyethylene matrix dissolution, i.e., its degradation.

The results showed that the presence of an aqueous environment had significant impacts on the physical, mechanical, thermal, and structural properties of the tested hybrid injection mouldings. The deterioration of their mechanical properties depended on the absorbed solvent type (plasticizing effect) and its quantity, the speed of liquid diffusion on the surface of the sample and inside it, the diffusion-related osmotic pressure inducing microcrack nucleation, as well as the hydrolytic degradation process.

This study has proved that pumpkin hulls can be used as natural fillers in the production of polymer composites. Nevertheless, it is equally important to maintain appropriate operating conditions in the production process.

## Figures and Tables

**Table 1:** Selected properties of Dowlex 2631.10UE.

Property	Value	Units
Density	935	kg m^−3^
Melt flow index (190 °C/2.16 kg)	7	g/10 min
Vicat softening point A120	115	°C
Deflection temperature under load HDT B	52	°C
Melting point	124	°C
Hardness, Shore D	56	°Sh
Tensile yield stress	17.8	MPa
Tensile strain at yield	419	%

**Table 2:** Surface roughness parameters of 20/0.8/LDPE samples before and after the solvent immersion.

Sample	Surface Roughness Parameters [µm]				
	*Ra*	*Rz*	*Rmax*	*Rq*	*RSm*
20/0.8/LDPE	1.48 ± 0.21	11.35 ± 0.03	22.55 ± 0.90	2.42 ± 0.11	0.147 ± 0.12
H_2_O/20/0.8/LDPE	2.03 ± 0.03	12.62± 0.79	31.23 ± 1.55	2.58 ± 0.79	0.377 ± 0.02
NaOH/20/0.8/LDPE	2.60 ± 1.02	15.98 ± 1.05	25.14 ± 3.83	3.39 ± 0.42	0.242 ± 0.07
C_3_H_6_O/20/0.8/LDPE	3.13 ± 0.21	16.92 ± 1.29	33.15 ± 3.53	3.57 ± 1.04	0.281 ± 0.08
C_7_H_8_O/5/0.8/LDPE	3.34 ± 0.58	20.33 ± 2.24	28.95 ± 1.37	4.26 ± 0.79	0.314 ± 0.04

## Data Availability

The data presented in this study are available on request from the corresponding author.
